# Adrenal cancer: relevance of different grading systems and subtypes

**DOI:** 10.1007/s12094-020-02524-2

**Published:** 2021-04-05

**Authors:** S. Minner, J. Schreiner, W. Saeger

**Affiliations:** 1 Institute of Pathology of the University of Hamburg, UKE, Martinistraße 52, 20246 Hamburg, Germany; 2 Clinic of Internal Medicine, Endocrinological Department of the University of Würzburg, 97080 Würzburg, Germany

**Keywords:** Adrenal, Cancer, Cancer types, Classification

## Abstract

**Purpose:**

The subclassification of adrenal cancers according to the WHO classification in ordinary, myxoid, oncocytic, and sarcomatoid as well as pediatric types is well established, but the criteria for each subtype are not sufficiently determined and the relative frequency of the different types of adrenal cancers has not been studied in large cohorts. Therefore, our large collection of surgically removed adrenal cancers should be reviewed o establish the criteria for the subtypes and to find out the frequency of the various types.

**Methods:**

In our series of 521 adrenal cancers the scoring systems of Weiss et al., Hough et al., van Slooten et al. and the new Helsinki score system were used for the ordinary type of cancer (97% of our series) and the myxoid type (0.8%). For oncocytic carcinomas (2%), the scoring system of Bisceglia et al. was applied.

**Results:**

Discrepancies between benign and malignant diagnoses from the first thee classical scoring systems are not rare (22% in our series) and could be resolved by the Helsinki score especially by Ki-67 index (more than 8% unequivocally malignant). Since all our cancer cases are positive in the Helsinki score, this system can replace the three elder systems. For identification of sarcomatoid cancer as rarest type in our series (0.2%), the scoring systems are not practical but additional immunostainings used for soft tissue tumors and in special cases molecular pathology are necessary to differentiate these cancers from adrenal sarcomas. According to the relative frequencies of the different subtypes of adrenal cancers the main type is the far most frequent (97%) followed by the oncocytic type (2%), the myxoid type (0.8%) and the very rare sarcomatoid type (0.2%).

**Conclusions:**

The Helsinki score is the best for differentiating adrenal carcinomas of the main, the oncocytic, and the myxoid type in routine work. Additional scoring systems for these carcinomas are generally not any longer necessary. Signs of proliferation (mitoses and Ki-67 index) and necroses are the most important criteria for diagnosis of malignancy.

## Introduction

According to the WHO classification 2017 of adrenocortical tumors [12], adrenal carcinomas are subdivided into main type, myxoid type, oncocytic type, and sarcomatoid type and a pediatric type. This subtyping is generally very important for the histological diagnosis of adrenal cancer, since the identification of malignancy is different for each subtype (Table 1). The malignancy of the main type and its myxoid subtype should be determined using the scoring system of Weiss et al. [39] supported by the scoring systems of van Slooten et al. [38] and of Hough et al. [15] or using the more simplified Helsinki score [29]. For oncocytic carcinomas, a special scoring system by Bisceglia et al. [4] has been established. The sarcomatoid adrenal cancers do not need a scoring system, since their malignancy is out of doubt. For pediatric cancers, the system of Wienecke et al. [40] should be used. The scoring systems are necessary for differentiation carcinomas from adenomas, but should be used only for specimens of adrenal resections and not for biopsies or metastases.

**Table 1:** Scoring systems for malignancy of adrenocortical carcinoma subtypes

Subtype	Scoring system
Main type	Weiss et al. [39]
	Van Slooten et al. [38]
	Hough et al. [15]
	Helsinki [29]
Myxoid	Weiss et al. [39]
	Van Slooten et al. [38]
	Hough et al. [15]
	Helsinki [15]
Oncocytic	Bisceglia et al. [4]
Pediatric	Wieneke et al. [40]
Sarcomatoid	Scoring system not necessary, malignancy absolutely certain

Detailed characteristics for identification of the subtypes are not found in the literature. Data of their frequency are sparse. In a series of 67 adrenal cancers, nine tumors were classified as oncocytic carcinomas [27]. Therefore, we reviewed our large collection of adrenal cancers for structural details in the subtypes and their relative frequency.

Aims of our studies are answers to the following questions:

1. Which scoring system is the best for the main type of adrenal cancers?
2. Are more than one scoring system necessary for identifying malignancy of the main type?
3. Which are the most important criteria for diagnoses of the different subtypes?
4. What are the relative frequencies of the different subtypes?

## Materials and methods

A collection of adrenal cancers from 521 patients treated between 1993 and 2005 were available for review. Pediatric carcinomas were not included. Paraffin-embedded specimens were sent together with some clinical data and macroscopic findings for second consultant diagnosis (WS).

Paraffin sections were stained with hematoxylin–eosin, PAS, and elastica-van Gieson, and additional immunostainings were performed: keratin Kl-1 or AE1/AE3, synaptophysin, chromogranin A, melan A, inhibin, SF-1, p53, and Ki-67.

The first step of our studies in each case was the assessment of possible cancer subtype. In the second step for assessing the malignancy, we used for the main subtype and the myxoid subtype the scoring system of Weiss et al. [39] (malignancy > 3) supported by the scoring systems of van Slooten et al. [38] (malignancy > 8) and of Hough et al. [15] (malignancy > 2.0). The Helsinki score [29] with the criteria necroses, mitoses, and Ki-67 index was additionally used for the final decision of malignancy if the three other scoring systems come to indeterminate levels between benign and malignant data. In oncocytic tumors, the system of Bisceglia et al. [4] was applied. Since pediatric tumors were not in our collection the scoring of Wieneke et al. [40] could not be used.

## Results

The following histological findings of the different subtypes base on our experiences from our large collection of adrenal cancers. Most of these descriptions are also found in textbooks of adrenocortical tumors [12, 21, 32].

### Main type

**Fig. 1:**
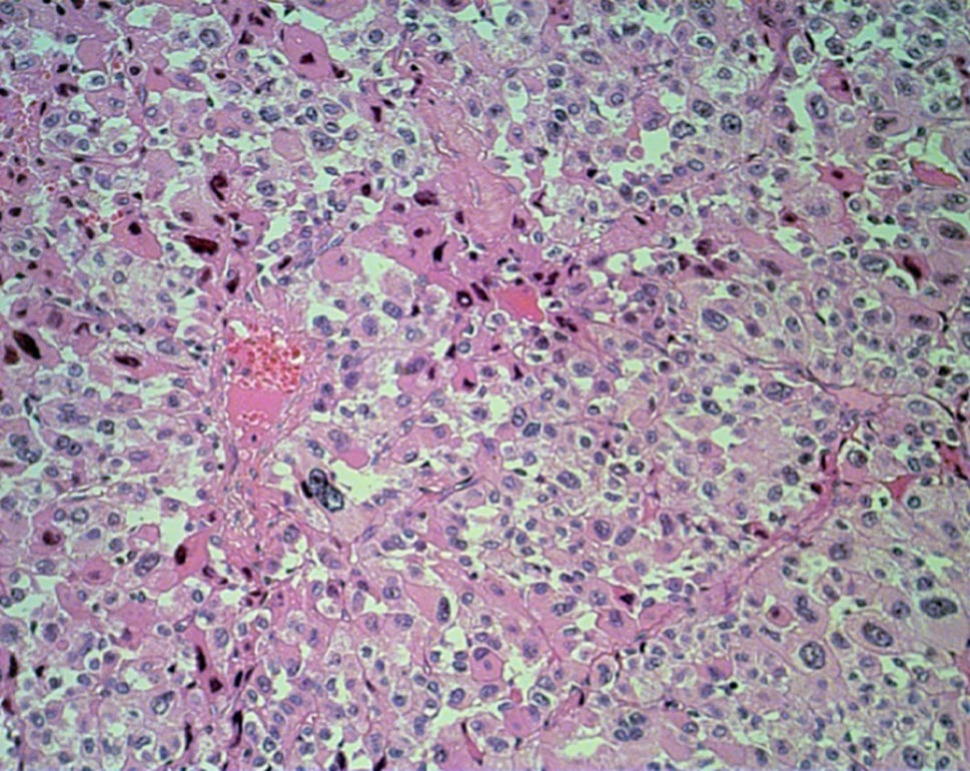
Main type with diffuse pattern, strong atypia, and thrombosis of sinusoidal vessel. Hematoxylin–eosin staining, 360×

The main type of adrenal cancer is characterized by diffuse growth pattern (Table 2) (Fig. 1). More solid type or nested, trabecular, or fascicular formations are rare. The intratumorous vessels are mostly of sinusoidal type with small endothelial layer and sparse or lacking muscular cell elements within thin walls. Circumscribed but present in more than two diameters of high power fields (HPF) or more extensive invasions of these vessels are often accompanied by thromboses that are followed by small or extensive, in necroses. The venous vessels in the periphery near the capsule can show circumscribed or multiple invasions. Partial of complete infiltrations of the fibrous capsules has to be noted. Fibroses may be present as an irregular network, but are more often found in larger fields. Some myxoid areas may be present, but should not exceed 40–50% of tumor volume. The tumor cells are slightly or strongly pleomorphic with increased chromatin. Nucleoli may be regular and small or irregular and increased. The number of mitoses is very different and has to be counted per high power fields. The cytoplasm shows mostly very few small lipid vacuoles or is free of visible lipid. Strong lipid accumulations or more differentiated spongiocytes as in normal adrenal cortex are rare.

**Table 2:** Growth pattern and proportions of adrenocortical carcinoma subtypes

Subtype	Growth pattern	Number	Proportion
Main type			
	Diffuse	471	
	Solid	26	
	Nested	4	
	Trabecular	3	
	Alveolar	1	
Sum		505	97%
Myxoid	Diffuse	3	
	Solid	1	
	Trabecular	1	
Sum		5	0.8%
Oncocytic	Diffuse	10	
Sum		10	2%
Sarcomatoid	Diffuse, fascicular	1	
			0.2%
All together		521	100%

In 29 (5.8%) of main type cancers, the index of Weiss et al., (malignancy > 3) was lower than four, but in 16 of these, the index of Hough et al., (malignancy > 2.0) was higher than 2.01. Of the remaining 13 cases, eight tumors showed a van Slooten et al., index (malignancy > 8) higher than eight. The 476 cancers with Weiss et al., score more than three (malignant) showed not malignant levels in the two other systems in 70 cases. In these, Hough et al., score was less than two (not malignant) in 62 cases and van Slooten et al., index lower than eight (not malignant) in 24 tumors. In five cancers with non-malignant data in all three scoring systems, the diagnosis of adrenal carcinoma is based on the Helsinki score with Ki-67 indexes of more than 8.5.

### Myxoid subtype

**Fig. 2:**
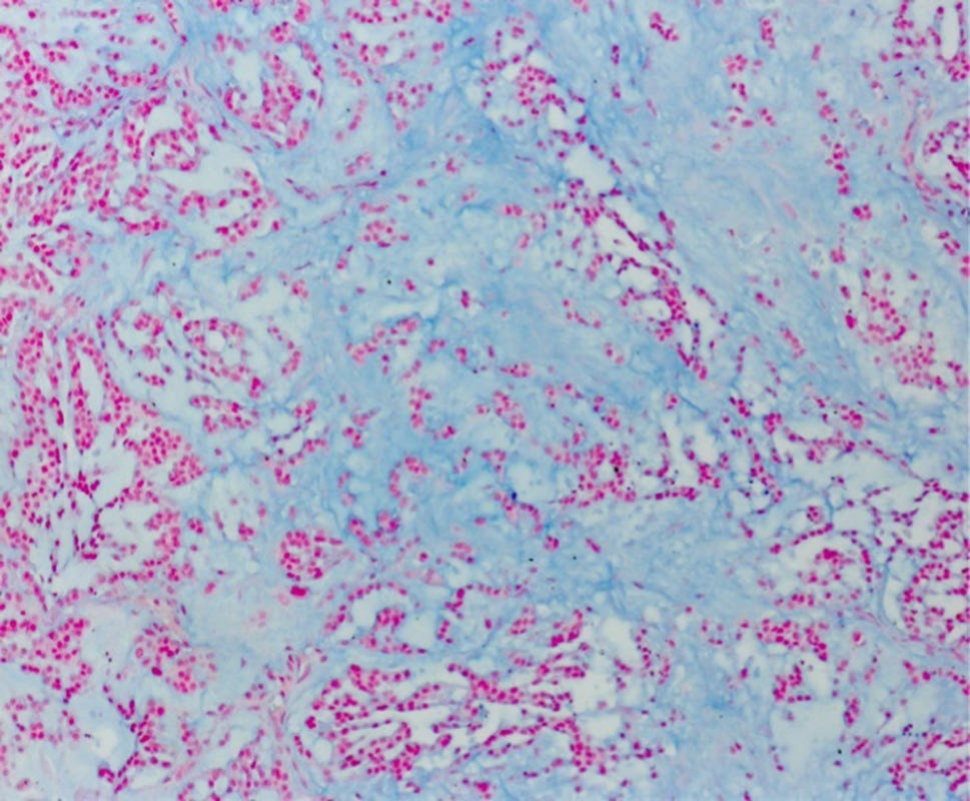
Myxoid type with strongly increased, Astra-blue myxoid stroma. Astra-blue staining, 220×

In our collection, we diagnosed a myxoid adrenal carcinoma if more than half of tumor volume was composed of myxoid matrix and the included tumor cells. When less than 50% we designate that as a main type cancer with myxoid parts. Otherwise, the myxoid subtype shows nearly all characteristics of the main type. The large areas of myxoid cell-free formations are strongly stained for alcian-blue (Fig. 2). The growth pattern is diffuse, solid, or trabecular (Table 2).

Oedematous areas are sometimes very similar and have to be differentiated from myxoid areas by negative staining for alcian-blue.

### Oncocytic subtype

**Fig. 3:**
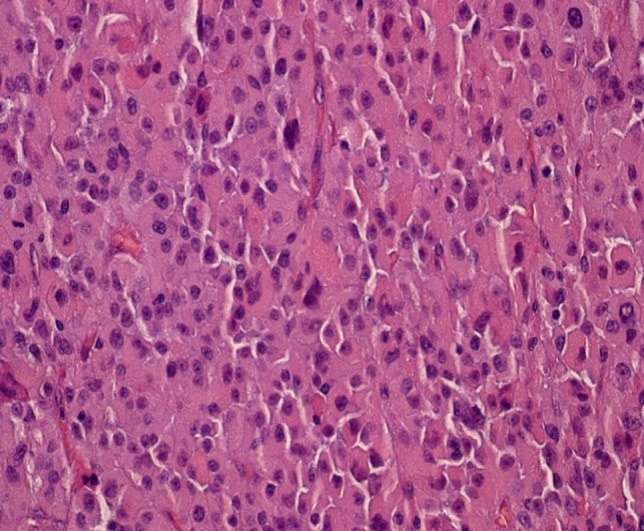
Oncocytic type with diffuse growth pattern, strong atypia, dense acidophilic cytoplasm, and no lipid vacuoles. Hematoxylin–eosin staining, 440×

The oncocytic subtype is always diffuse in architecture (Table 2) (Fig. 3). The cells can be very pleomorphic harboring nuclei with strongly increased chromatin und irregular nucleoli. The cytoplasm has to be strongly eosinophilic with diffuse dense staining. Lipid vacuoles are lacking. All other criteria are similar to the main type. The substantial criteria for differentiation from oncocytic adenomas are an increased mitotic index (> 5/50 HPF), atypical mitoses, and invasion of veins. One main criterion is enough or sufficient for diagnosis of malignancy. Minor important criteria are size (more than 100 mm in diameter) and weight of tumor (> 200 g), invasion of sinusoidal vessels and capsule, and necroses.

### Sarcomatoid subtype

**Fig. 4:**
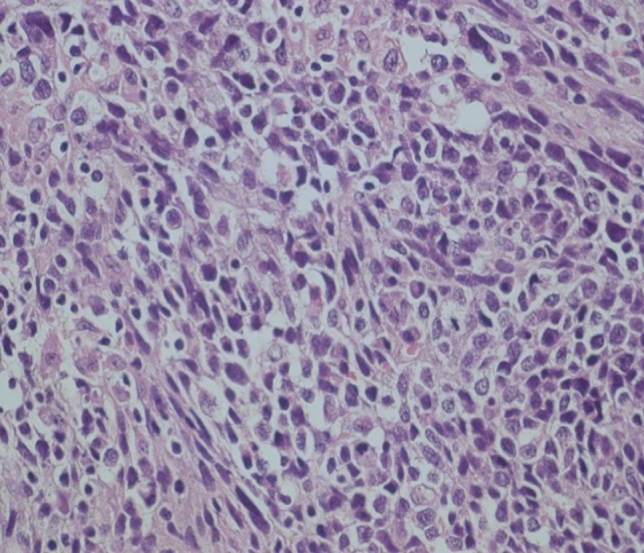
Sarcomatoid type with outlined fascicular pattern, spindly pleomorphic cells with atypical nuclei. Hematoxylin–eosin staining, 360×

The sarcomatoid subtype is characterized by a mostly very irregular, often partly spindle cell-like epithelial component and by sarcomatoid mesenchymal formations with spindle cell or fascicular formations (Table 2) (Fig. 4). For the differentiation of the sarcomatoid part, immunostainings are essential. By these, rhabdomyoblastic, leiomyoblastic, fibroblastic, lipoblastic, chondroid, or osteoid areas can be identified. In some cases, additional methods of molecular pathology are necessary.

### Comparison of scoring systems

For evaluation of special importance of different criteria in the four scoring systems (Table 3), we gathered the criteria into the groups growth pattern, cell structures, proliferation parameters, proportion of spongiocytes, invasion of vessels or capsule, and regressive changes. The diffuse growth pattern is very important only in the system of Hough et al. [15]. The atypical cell structures (nuclear atypia, nuclear hyperchromasia, and enlarged nucleoli) are most important in the system of van Slooten et al. [38]. If all of these criteria for atypical cell structures are present, the threshold value is exceeded. In two other systems, these structures are much lower important reaching a value of up to 19% for reaching the lowest value for diagnosing malignancy. The signs of proliferation (mitoses) are differently important: in the system of van Slooten et al. [38], the value for malignancy can be fulfilled by one criterion of increased proliferation (mitoses in > 2/10 HPF). The Helsinki score is the only one taking the Ki-67 index into account (value three or more than 5/50 mitoses per HPF plus the number of Ki-67 index). Regressive changes are very important in the systems of Van Slooten et al. score [38] (value 70%) and Hough et al. score [15] (value 33%) and most important in the Helsinki score. Since all cancers of our collection showed a number of more than the threshold value in the Helsinki score [29], this system appeared to be the most reliable procedure—the gold standard—in differentiating adrenal carcinomas from cortical adenomas.

**Table 3:** Assessments of the different criteria in the scoring systems

Criteria	Weiss et al. score [39]	Van Slooten et al. score [38]	Hough et al. score [15]	Helsinki score [29]
Growth pattern	1 (11%)	0 (0%)	0.92 (46%)	0 (0%)
Diffuse growth pattern	1		0.92	
Cell structures	1 (11%)	8.8 (110%)	0.39 (19%)	
Nuclear atypia	1	2.1	0.39	
Nuclear hyperchromasia		2.6		
Enlargement of nucleoli		4.1		
Proliferation	2 (22%)	9 (112%)	0.60 (33%)	3 (35%)
Mitoses > 5/50 HPF	1			3
Mitoses > 10/00 HPF			0.60	
Mitoses > 2/10 HPF		9		
Atypical mitoses	1			
Ki-67 index				Number
Spongiocytes < 21%	1 (11%)	0 (0%)	0 (0%)	0 (0%)
Invasion	3 (33%)	3.3 (41%)	1.29 (64%)	
Of capsule	1		0.37	
Of sinusoidal vessels	1			
Of veins	1		0.92	
Of vessels or capsule		3.3		
Regressive lesions	1 (11%)	5.7 (71%)	1.69 (64%)	5 (59%)
Necroses > 2 HPF	1		0.69	5
Necroses and fibroses		5.7		
Broad fibroses			1.0	
Lowest value for malignancy	> 3 (100%)	≥ 8 (100%)	> 2.0 (100%)	> 8.5 (100%)

The numbers in brackets indicate the proportion for reaching the lowest value for diagnosing malignancy

### Relative frequencies of subtypes

Our data of subtyping show that the main type comprehends 97% of all adrenocortical cancers, the oncocytic type 2%, the myxoid type 0.8%, and the rarest sarcomatoid type 0.2% (Table 2).

## Discussion

### Main type

From our studies, the most characteristic features of the main type are the signs of increased proliferation (mitoses > 5/50 HPF, Ki-67 index > 5%) and the amount of necroses (diameter > 2 HPF). Less important criteria are the growth pattern, the infiltration of capsule and vessels, and the amount of lipid vacuoles in the cytoplasm.

The histomorphology, immunostaining, and differential diagnosis of the adrenocortical main type cancer have been published in the WHO classification of 2004 [23] and 2017 [12] but also in other textbooks [8, 21] and are reflected by the scoring systems of Weiss et al. [39], van Slooten et al. [38], and of Hough et al. [15] and by the Helsinki score [29]. All these characteristics correspond to our findings in our large collection.

Discrepancies between benign and malignant diagnoses from data of the classical three scoring systems lead to the diagnosis “indeterminate malignancy”. This could be canceled by the Helsinki score [29] by the criteria necroses, mitoses, and (most important) Ki-67 index more than eight. Therefore, it should be discussed whether or not the Helsinki score can replace the classical three scoring systems and be used alone.

### Myxoid subtype

Our criterion for the myxoid subtype was the enormous amount of myxoid fields with alcian bue-positivity going beyond half of tumor volume. Cancers with smaller myxoid areas were assigned to the main type as all other criteria are identical with the main type.

All reported myxoid adrenal tumors are endocrine active. About half of them are benign [16, 31]. The areas of myxoid alterations comprehend between 5 and 90% of tumor volume [28].

Differences of myxoid cancers in their prognosis compared with the main type carcinomas could not be found [28].

### Oncocytic subtype

In contrast to the main type, the oncocytic cancer type is more complicated and controversially discussed in the literature. Only about 20% of adrenocortical oncocytic tumors are malignant [19, 26]. To assess malignancy, Bisceglia et al. [4] created a special scoring system with mayor criteria (Mitotic rate of more than 5 per HPF, atypical mitotic figures, or venous invasion). Only one of these main criteria should be sufficient for diagnosis of malignancy, whereas other criteria (nuclear pleomorphism, nucleoli) of other scoring systems are completely irrelevant. In our opinion, oncocytic tumors are nearly entirely composed of oncocytes, whereas others [4] define them by oncocytic predominance. We agree with Bisceglia et al. [4], Mearini et al. [26], and Summer et al. [36] that oncocytic adrenocortical tumors are hormonally inactive probably due to the strongly increased but malfunctioning mitochondria. Others have reported that between 10 and 20% of oncocytic adrenocortical tumors produce hormones [42] and one case report was published in which Cushing’s syndrome was induced by this tumor type [2]. We believe that these are not oncocytic active tumors but adrenocortical tumors especially of lipid-poor compact cell type that are more active than lipid-rich cells. The differentiation from oncocytic tumors may be possible when a minority of tumor cells show lipid vacuoles in the cytoplasm which are lacking in oncocytic tumors. In animal experiments, it could be shown that increased stimulation of adrenocortical cells induces a reduction of spongiocytes and an increase of compact cells [33].

### Sarcomatoid subtype

If an adrenocortical malignant tumor shows areas of epithelial differentiation and greater areas of sarcomatous differentiation, the term “carcinosarcoma” [11, 20, 22, 37] should be used. If a more homogenous tumorous proliferation possesses some characteristics of sarcomas and some of carcinomas, the term “sarcomatoid carcinoma” [6, 10, 14, 18, 18, 24, 35] is appropriate. The sarcomatous features including their immunostainings have similarities with leiomyosarcoma [5], rhabdomyosarcoma [7, 11, 37], spindle cell sarcoma [5, 11, 22, 35], or osteosarcoma [3].

One tumor showed remnants of adrenocortical features—a precursor lesion?—in an otherwise dedifferentiated sarcoma [34]. If no adrenocortical features could be found, an adrenal sarcoma is likely [13, 17, 25]. Immunostainings are generally necessary for differentiation and, in single cases, molecular pathology [34].

### Comparison of scoring systems

The four scoring systems (Table 3) have different priorities. The Weiss score [39] stresses the invasion of capsule and vessels (3 points), the van Sooten score [38], the atypical cell structures, and the mitotic index, the Hough score [15], the atypical cell structures, the regressive changes, and the diffuse growth pattern.

The Helsinki score [29] based on studies of 167 consecutive adrenal cancers that were identified with the Weiss score and clinical follow-up checks. By integrating the Ki-67 index in the differentiation of carcinomas from adenomas, the authors developed their system which showed a sensitivity of 100% and a specificity of 99.6%.

In our studies, all adrenal cancers showed a scoring number of more than the threshold value of 8.5 in the Helsinki score. Therefore, we can confirm the data of Pennanen et al. [29] and emphasize that the Helsinki score is the most reliable procedure in differentiating adrenal carcinomas from cortical adenomas.

### Molecular pathology

Molecular pathology became very important for choice of treatment and prognosis of malignant tumors, but for adrenal cancers, tumor programmed death-ligand 1 (PD-L1) expression and MSI-H/MMR-D status were not associated with objective response [30] Adrenocortical carcinoma is a Lynch syndrome-associated cancer. Three of four patients carried a pathogenic germ-line mutation in a mismatch repair gene [1]. Whether or not these molecular alterations correlate to subtypes of adrenal cancer is not known and should be studied.

### Prognosis

The median overall prognosis amount to 3–4 years [9]. 5 year survival comes to 60–80% if the tumor is confined to adrenal space, to 35–50% if the cancer is locally advanced, and to 0–25% if metastases exist [9]. Further important prognostic markers are Ki-67 index, Weiss score, mitotic index, R-status [9], and chromosomal aberrations [41]. An adjuvant mitotane therapy is necessary if Ki-67 index exceeds 10%, and an advanced stage or an R1 status exists [9].

### Statistic

The statistical data from our large collection concerning the relative frequency of oncocytic adrenal cancers (2%) are in strong contrast to data (22%) found by others [27]. The main type is in the huge majority of our collection, and the subtypes comprehend 3% of all adrenal cancers.

## Conclusion

Answering the questions in introduction, we confirm

1. that the Helsinki score is the best for differentiating adrenal carcinomas of the main and myxoid type in routine work,
2. that additional scoring systems for these carcinomas are generally not any longer necessary,
3. that signs of proliferation (mitoses and Ki-67 index) and necroses are the most important criteria for diagnoses of the different subtypes,
4. that, according to the relative frequencies of the different subtypes of adrenal cancers, the main type is the far most frequent (97%) followed by the oncocytic type (2%), the myxoid type (0.8%), and the very rare sarcomatoid type (0.2%).
